# Supplementary material for: Deep learning predicts real-world electric vehicle direct current charging profiles and durations
Source: Nat Commun. 2025 Dec 5;16:10921. doi: 10.1038/s41467-025-65970-y (PMC12680626; doi:10.1038/s41467-025-65970-y)
Supplement: Supplementary file 1 — Supplementary Information [file 41467_2025_65970_MOESM1_ESM.pdf]

Supplementary Information: Artificial intelligence predicts real-world EV DC charging profiles and durations

Siyi Li[1*], Mingrui Zhang[1], Robert Doel[2], Benjamin Ross[2], Matthew D. Piggott[1]

[1]Department of Earth Science and Engineering, Imperial College London, London, SW7 2AZ, UK.
[2]Shell Research Limited, London, SE1 7NA, UK.

*Corresponding author. E-mail: siyi.li20@imperial.ac.uk;

Contents

S1 TFT achitecture details **3**

S2 Training and validation losses for the TFT model **4**

S3 Distribution analysis on held-out test sets **5**

S4 Extended performance evaluation **7**

S5 Charging profile prediction model inference time **8**

S6 Impact of static covariates on the performance of the charge curve prediction model **9**

S1 TFT achitecture details

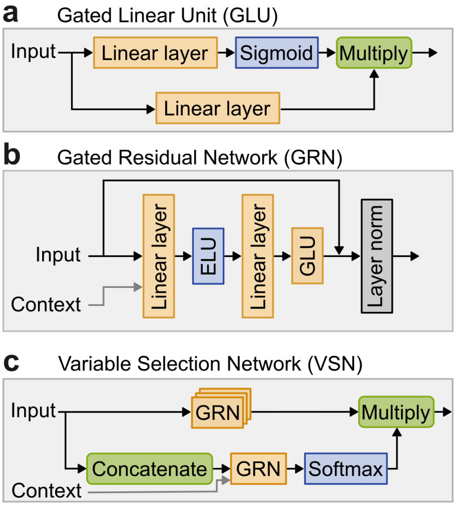

Supplementary Figure 1 Detailed neural network modules including Gated Linear Unit (GLU), Gated Residual Network (GRN) as well as Variable Selection Network (VSN). ELU standards for Exponential Linear Unit. Together these make up key architectural components of the anomaly detection and charging profile prediction models.

S2 Training and validation losses for the TFT model

Supplementary Figure 2 shows the training and validation loss curves for the TFT model from three separate training runs, each initialised with a different random seed. The differing seeds resulted in variations in weight initialisation, data shuffling, and trainvalidation splits. All models were trained to convergence using a reduce-on-plateau learning rate scheduler, which lowered the learning rate when improvements in validation loss plateaued. Model selection was based on the sum of validation losses across the different input point levels.

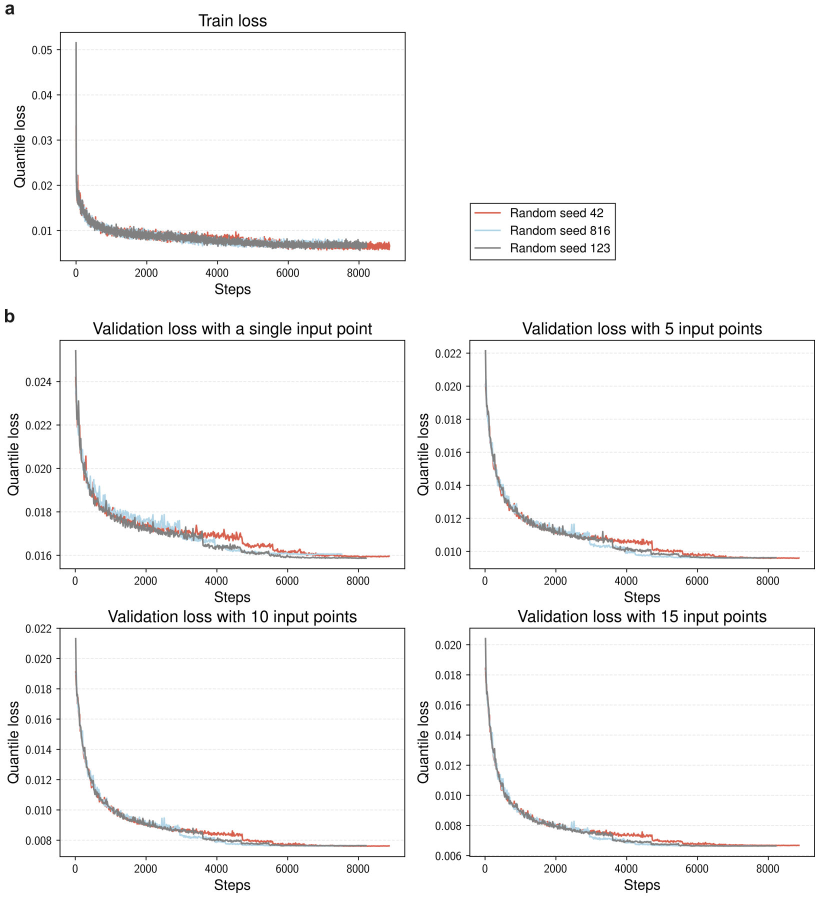

Supplementary Figure 2 Training and validation loss curves of the charging profile prediction model with the TFT architecture. The model was trained using the quantile loss with three random seeds with different random initialisation and data shuffling. **a** Training losses where a random portion of the charging curves are masked for training. **b** Validation losses with model performance evaluated using fixed input point counts of 1, 5, 10, and 15.

S3 Distribution analysis on held-out test sets

To assess whether the held-out test sets for the charging profile prediction model differ meaningfully from the training data in distribution, a permutation-based statistical test was conducted using the 1-Wasserstein distance as a distributional divergence metric.

For each dataset pair, the observed 1-Wasserstein distance was computed between the training and test sets in the latent space of the trained β-VAE model. Distances were first calculated independently for each latent dimension, and the average across all dimensions was used as the overall test statistic. The 1-Wasserstein distance quantifies the minimum effort required to morph one probability distribution into another and serves as an interpretable proxy for distributional similarity. Notably, the β-VAE was trained on a random train/test split that differs from the one used for the main charging profile prediction model, ensuring independence between these processes. To evaluate statistical significance, a permutation test with 1,000 iterations was performed under the null hypothesis that both datasets are drawn from the same distribution. In each iteration, all latent vectors from the training and test sets were combined and randomly partitioned into two groups of equal size. The mean 1-Wasserstein distance was computed for each pair, forming a null distribution of distances expected under random assignment. The empirical p-value was calculated as the proportion of permuted distances greater than or equal to the observed distance. The results are illustrated in Supplementary Figure 3.

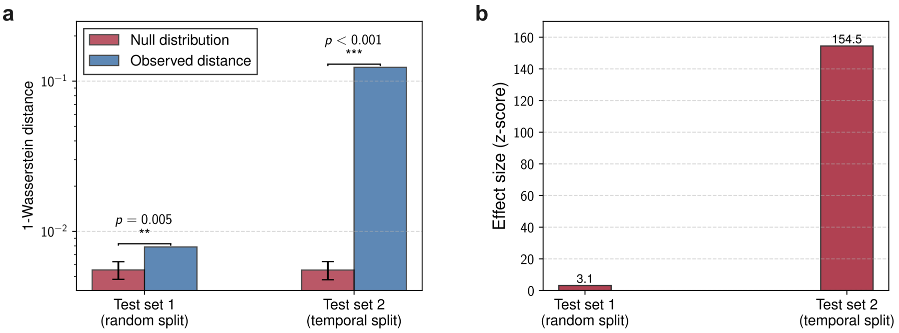

Supplementary Figure 3 **a** 1-Wasserstein distance between train and test sets shows higher observed values than the null, especially for temporal splits ($p < 0.001$). Bars show the mean of the null distribution with error bars indicating ± one standard deviation (SD). **b** Effect size measured with z-score is minimal for test set one with random split but large for test set two with temporal splits.

While the permutation-based 1-Wasserstein test identified a statistically significant difference between the training set and test set one (random split), the observed effect size is modest ($z = 3.1$). Given the large sample sizes involved, with over 700,000 sessions in the training set and 45,000 in each test

set, even minimal variation between distributions can yield low p-values due to high statistical power. It is also possible that projection onto the latent space amplified the subtle differences in distributions. By contrast, the temporal split (test set two) exhibits a much more pronounced deviation from the training distribution ($z = 154.5$, $p < 0.001$), consistent with a genuine distributional shift, as these sessions were drawn from a later time period not covered during training.

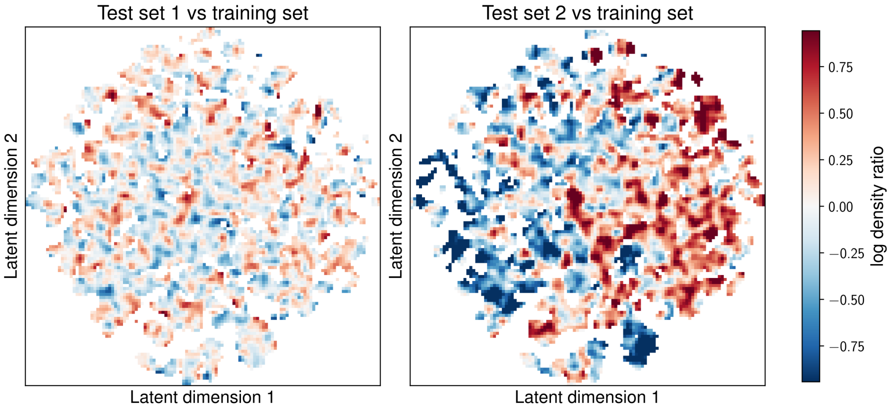

Supplementary Figure 4 t-SNE latent space density ratio maps comparing each test set with the training set under a shared colour scale. Colour intensity reflects the magnitude of the relative density difference with respect to the training set; hue encodes direction (blue indicates under-representation in the test split, red indicates over-representation). **Left:** test set one vs training shows mostly small, diffuse deviations near zero. **Right:** test set two vs training shows coherent regions of deficit and pockets of excess, consistent with a temporal distribution shift.

To complement the permutation analysis, distributional differences in the β-VAE latent space were visualised with t-SNE density ratio maps and shown in Supplementary Figure 4. Latent vectors from the training set and from each test sets were embedded to two dimensions with t-SNE. Densities were estimated on a common grid and lightly smoothed. For each bin the log ratio

$$\log\left(\frac{\text{density}_{\text{test}}}{\text{density}_{\text{train}}}\right)$$

was computed, and bins with insufficient support were masked. A single symmetric colour scale was applied across both panels.

S4 Extended performance evaluation

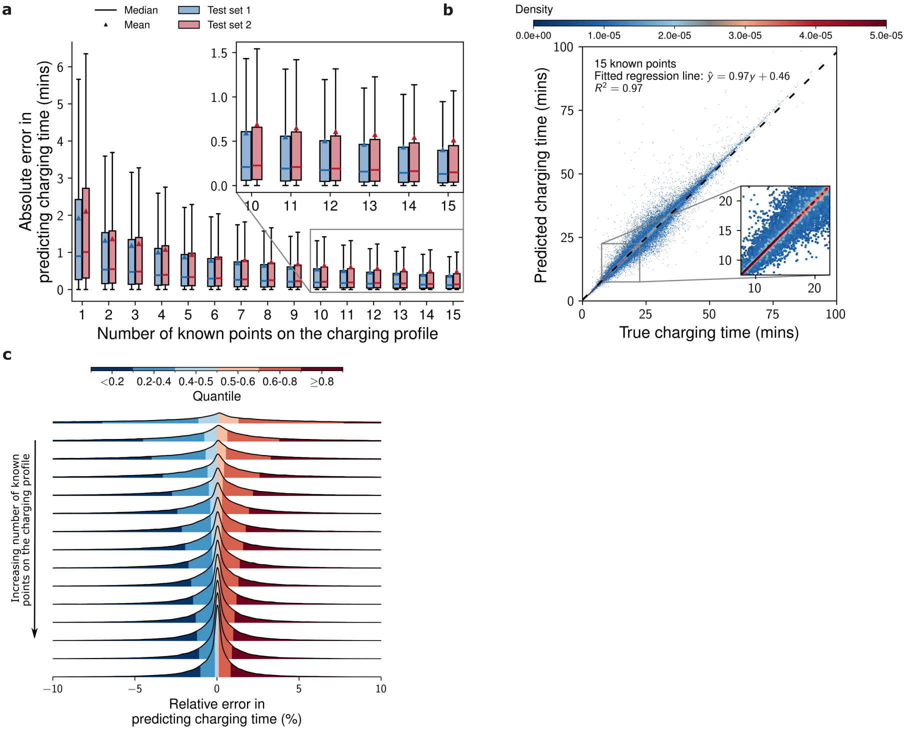

Supplementary Figure 5 Extended performance evaluation of the charging profile prediction model, where the model's median predictions were compared to ground truth charging profiles and times. Charging times were calculated based on either the completion of the charging session or reaching 80% SoC, whichever came first. Analysis was confined to sessions with a strictly greater than 15% change in SoC. **a** The charging profile prediction model's performance on predicting charging time for different numbers of known points of the charging profile as input, evaluated on the two test sets using absolute error. Box plots show the median (centre line), mean (triangles), interquartile range (box limits), and whiskers (1.5× the interquartile range). **b** Scatter plot with density overlays comparing median predicted charging times against ground truth when 15 points were used as input The results combine data from both test sets. **f** Distribution analysis of relative errors in predicting charging time, across varying numbers of input points on the charging profile, incorporating results from both test sets.

S5 Charging profile prediction model inference time

Inference benchmarks on the charging profile prediction model are conducted on a dedicated compute node configured with an AMD EPYC 7742 64-core processor and a single 40 GB NVIDIA A100 GPU. The benchmarking evaluates the inference efficiency of the model under varying batch sizes, comparing CPU-only and GPU-accelerated execution timing, with the timing statistics reported over 50 runs. The results are displayed in Supplementary Table 1.

Supplementary Table 1 Forward pass timing and throughput across batch sizes on CPU and GPU.

**Batch Size**	CPU Time (ms)	GPU Time (ms)	CPU Throughput (samples/sec)	GPU Throughput (samples/sec)
1	37.17 ± 0.46	12.13 ± 0.04	26.9	82.4
16	309.91 ± 3.83	12.72 ± 0.02	51.6	1257.5
32	625.27 ± 28.35	13.24 ± 0.06	51.2	2416.2
64	1302.49 ± 42.44	15.39 ± 0.04	49.1	4158.0
128	2759.00 ± 55.99	20.36 ± 1.58	46.4	6287.4
256	5596.13 ± 82.89	32.63 ± 0.11	45.7	7844.4
512	12126.96 ± 130.86	54.69 ± 0.20	42.2	9361.8
1024	24577.94 ± 117.24	101.08 ± 0.26	41.7	10130.7

S6 Impact of static covariates on the performance of the charge curve prediction model

The performance of the models trained with different configurations of static covariates are shown in Supplementary Figure 6. It is evident that the full model achieved the highest accuracy in predicting charging profiles and charging times. Models trained without estimated capacity exhibited a noticeable reduction in accuracy, while models trained without ambient temperature showed a slight decrease in accuracy, particularly when the number of known points on the charging profile was low. However, as the number of known points increases, the differences in accuracy among the models become smaller. This outcome aligns with the observed training and validation losses and can be explained by the shift in importance from static covariates to the time series data as more points on the charging profile become available. It is worth noting that the two static covariates had a much smaller impact on the anomaly detection model, as its performance primarily depended on the time series features. Consequently, similar experiment results for the anomaly detection model were not included, as the variation in accuracy across different configurations of static covariates was negligible.

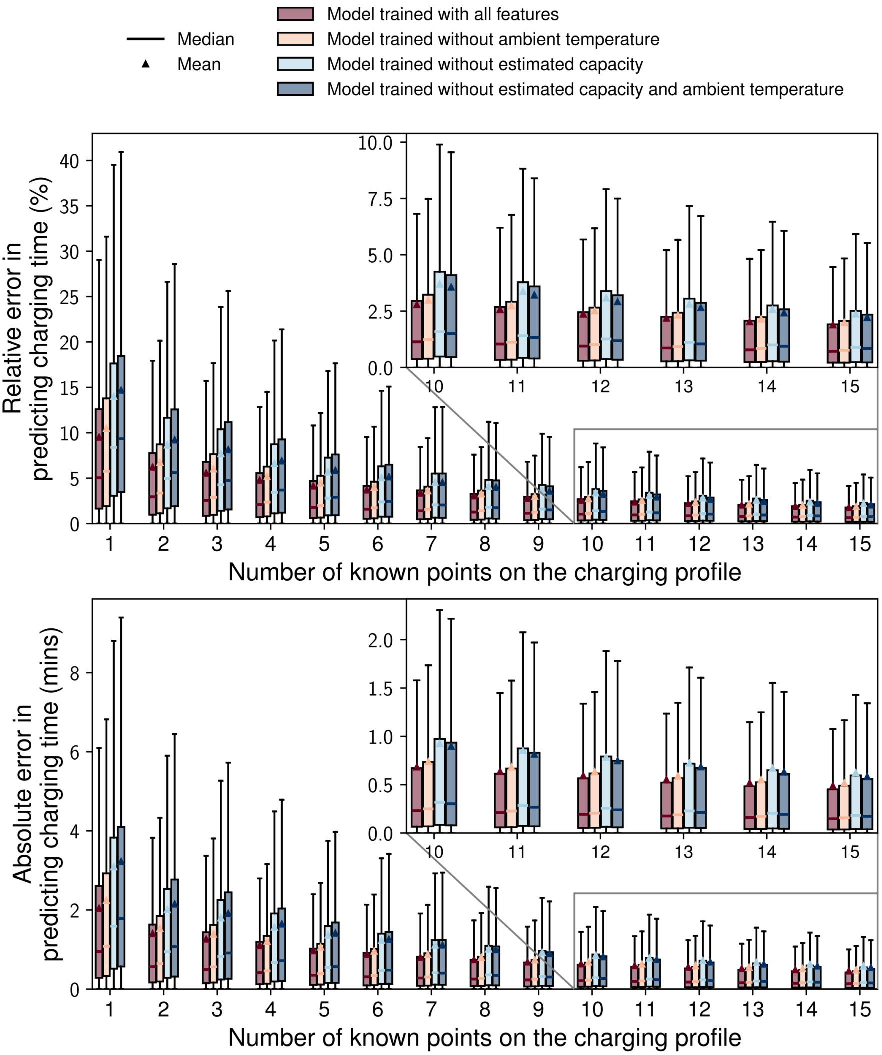

Supplementary Figure 6 Performance evaluation of the charging profile prediction model trained with different configurations of static covariates. Charging times were calculated based on either the completion of charging or reaching 80% SoC, whichever came first. The results integrate data from both test sets. Box plots show the median (centre line), mean (triangles), interquartile range (box limits), and whiskers (1.5× the interquartile range).
